# First Recombinant High-Density Lipoprotein Particles Administration in a Severe ICU COVID-19 Patient, a Multi-Omics Exploratory Investigation

**DOI:** 10.3390/biomedicines10040754

**Published:** 2022-03-23

**Authors:** Sébastien Tanaka, Floran Begue, Bryan Veeren, Alexy Tran-Dinh, Tiphaine Robert, Parvine Tashk, Brice Lortat-Jacob, Dorothée Faille, Luc de Chaisemartin, Nathalie Zappella, Enora Atchade, Laura Kramer, Philippe Montravers, Olivier Meilhac

**Affiliations:** 1 AP-HP, Service d’Anesthésie-Réanimation, CHU Bichat-Claude Bernard, 75018 Paris, France; alexy.trandinh@aphp.fr (A.T.-D.); parvine.tashk@aphp.fr (P.T.); brice.lortat-jacob@aphp.fr (B.L.-J.); nathalie.zappella@aphp.fr (N.Z.); enora.atchade@aphp.fr (E.A.); philippe.montravers@aphp.fr (P.M.); 2 INSERM, UMR 1188 Diabète Athérothombose Réunion Océan Indien (DéTROI), Université de La Réunion, 97400 Saint-Denis, France; floran.begue@univ-reunion.fr (F.B.); bryan.veeren@univ-reunion.fr (B.V.); 3 Institut National de la Santé et de la Recherche Médicale (INSERM), Université de Paris, 75006 Paris, France; dorothee.faille@aphp.fr; 4 INSERM U1148, Laboratory for Vascular Translational Science, 75018 Paris, France; 5 AP-HP, Service de Biochimie, CHU Bichat-Claude Bernard, 75018 Paris, France; tiphaine.robert@aphp.fr; 6 AP-HP, Département d’Hématologie Biologique, CHU Bichat-Claude Bernard, 75018 Paris, France; 7 AP-HP, Département d’Immunologie, CHU Bichat-Claude Bernard, 75018 Paris, France; luc.de-chaisemartin@u-psud.fr; 8 Inserm, Inflammation, Microbiome, Immunosurveillance, Université Paris-Saclay, 92290 Châtenay-Malabry, France; 9 AP-HP, Service de Pharmacie, CHU Bichat-Claude Bernard, 75018 Paris, France; laura.kramer@aphp.fr; 10 INSERM UMR 1152—PHERE, Physiopathology and Epidemiology of Respiratory Diseases, 75018 Paris, France; 11 CHU de La Réunion, CIC-EC 1410, 97400 Saint-Denis, France

**Keywords:** COVID-19, lipoprotein, HDL, proteomics, CER-001, case-report, sepsis, cytokines, inflammation

## Abstract

High-density lipoproteins (HDLs) have multiple endothelioprotective properties. During SARS-CoV-2 infection, HDL-cholesterol (HDL-C) concentration is markedly reduced, and studies have described severe impairment of the functionality of HDL particles. Here, we report a multi-omic investigation of the first administration of recombinant HDL (rHDL) particles in a severe COVID-19 patient in an intensive care unit. Plasma ApoA1 increased and HDL-C decreased after each recombinant HDL injection, suggesting that these particles were functional in terms of reverse cholesterol transport. The proportion of large HDL particles also increased after injection of recombinant HDL. Shotgun proteomics performed on HDLs isolated by ultracentrifugation indicated that ApoA1 was more abundant after injections whereas most of the pro-inflammatory proteins identified were less abundant. Assessment of Serum amyloid A-1, inflammatory markers, and cytokines showed a significant decrease for most of them during recombinant HDL infusion. Our results suggest that recombinant HDL infusion is feasible and a potential therapeutic strategy to be explored in COVID-19 patients.

## 1. Introduction

High-density lipoproteins (HDL) belong to a family of particles characterized by their ability to transport cholesterol from peripheral tissues to the liver, which confers them cardiovascular protective effects [1,2]. In addition to their reverse cholesterol transport function, HDLs exhibit pleiotropic endothelioprotective properties, including anti-inflammatory, anti-apoptotic, anti-thrombotic, and antioxidant functions [3,4,5,6,7,8]. HDLs also have the ability to neutralize lipopolysaccharide (LPS) and promote its clearance, giving them anti-infectious effects [9,10,11]. During acute inflammatory states, recent clinical studies have highlighted the fact that HDL-cholesterol (HDL-C) levels are low [12,13,14,15,16] and HDL particles are qualitatively altered leading to their dysfunction [17,18,19,20,21], which prevent them from exerting their protective functions in pathologies such as sepsis where endothelial function is highly compromised. In this context, preclinical studies have investigated the interest of injecting recombinant or reconstituted HDLs in animal models of sepsis [22,23,24,25,26,27,28]. These studies have highlighted the interest of these injections with a drastic reduction in mortality, an improvement in organ dysfunctions and inflammation markers, making it possible to envisage future clinical trials in septic patients [29].

Coronavirus 2019 disease (COVID-19), which is caused by severe acute respiratory syndrome coronavirus 2 (SARS-CoV-2), was first detected in December 2019, declared a global pandemic in March 2020, and has caused more than 5 million deaths worldwide [30,31]. Several studies, including ours [32], have reported significant changes in the lipid profile of COVID-19 patients.

As in bacterial sepsis, HDL-C concentration is low during COVID-19 and several studies have shown a correlation between this low HDL-C level and morbi-mortality [32,33,34,35,36]. In a recent study in severe COVID-19 patients compared to controls, our team showed major changes in the HDL proteome with, in particular, less apolipoprotein A-I and paraoxonase 1 and more pro-inflammatory proteins such as serum amyloid A (SAA-1 and 2) [37]. In addition, HDLs from patients were less protective in TNFα-stimulated endothelial cells and inhibition of apoptosis by HDL isolated from COVID-19 patients was btunted relative to HDLs from controls. Recently, in a pilot study involving COVID-19 patients, NMR spectroscopy was used and showed a reduced number of HDL particles, in particular, a low number of small functional HDL particles [38]. Overall, the HDL profile of COVID-19 patients appears to be quite similar to that of patients with bacterial sepsis with a drastic decrease in HDL-C concentration, changes in HDL proteome and size, and impaired protective effects. These data support the potential of administering functional HDL particles in COVID-19 patients. Several pharmaceutical companies have developed apoA1 nanoparticles reconstituted from recombinant ApoA1 or ApoA1 isolated from human plasma in combination with phospholipids (rHDL). Despite disappointing effects on cardiovascular disease [39,40,41], these rHDL particles were found to be safe when intravenously injected to patients [42,43] and display antioxidant and anti-inflammatory properties similar to those of plasma-isolated HDLs [44,45]. Recently, intravenous administration of rHDLs in patients with familial lecithin–cholesterol acyltransferase (LCAT) deficiency, has shown very promising renal and ophthalmologic results [46,47].

Here we present an exploratory multi-omics study of the first intravenous administration of rHDL particles in a severe COVID-19 patient in an intensive care unit (ICU).

## 2. Case Report

### 2.1. Clinical Presentation of the Case Report

A 66-year-old woman with overweight, hypothyroidism, and chronic obstructive pulmonary disease, was hospitalized in ICU for severe COVID-19 requiring high-flow oxygen therapy alternating with noninvasive ventilation on admission. Corticosteroids were administered daily from admission to the ICU for seven days. Because the clinical course was rapidly unfavorable, with, in particular, severe hypoxemia, compassionate administration of recombinant HDL particles (rHDL, CER-001, Abionyx Pharma, Labège, France) was initiated on the first day with a temporary authorization for use (authorization number 58489 from the French National Agency for the Safety of Medicines and Health Products) and after informed consent. Abionyx Pharma did not have access to the data during the treatment period and was not involved in the drafting of the manuscript or the decision to submit it for publication. rHDLs were injected intravenously every 12 h for 3 consecutive days after ICU admission. EDTA plasma was collected before (H0, H12) and 3 h after rHDL injection (H3 and H15). Additional plasma samples were obtained on Days 4, 5, 6, and 7. Laboratory tests at admission and during the patient’s hospitalization in the ICU are described in Table 1. Despite relative stability of the patient’s respiratory function during CER-001 treatment with high-flow oxygen therapy and noninvasive ventilation, the patient inexorably deteriorated on day 4, requiring intubation on day 5. The evolution was marked by a severe acute respiratory distress syndrome motivating the administration of a neuromuscular blocking agent and prone positioning without a positive effect on oxygenation. The evolution was unfavorable, and the patient died on Day 7.

### 2.2. Lipid Profile after rHDL Injections

**Figure 1:**
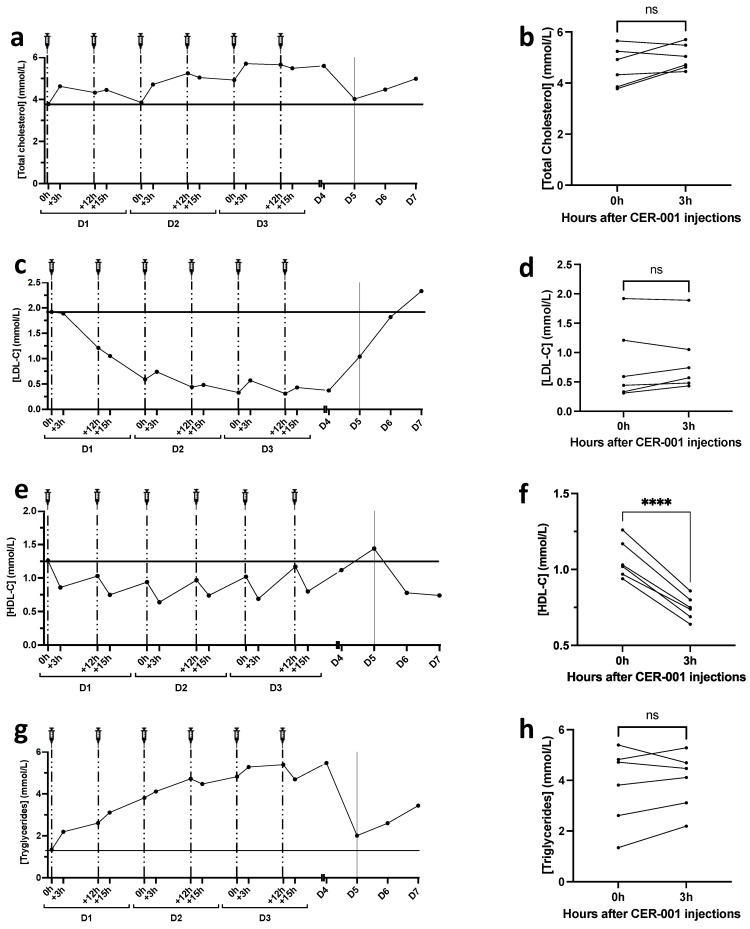
Changes in total cholesterol (TC), LDL-C, HDL-C, and triglyceride profiles during CER-001 treatment. TC, LDL-C, HDL-C, and TG were determined using an enzyme kit (CHOL, HDL-C, LDL-C, TRIG methods, Dimension VISTA System, Siemens Healthineers) at all time points, entire methods are exposed in Supplementary File S1. The patient received two injections of CER-001 per day for three days with a 12 h interval between each injection represented by the syringes and dotted lines. On day 5, the patient entered a critical phase represented by a solid line at D5. Plasma was collected in EDTA tubes at different times: 0 h: before CER-001 injection; +3 h: 3 h after the first injection; +12 h: 12 h after the first injection and before the second injection; +15 h: 15 h after the first injection and 3 h after the second injection. (**a**) Total cholesterol levels during the treatment period before and 3 h after each CER-001 injection (**b**). (**c**–**f**) LDL-C and HDL-C levels during CER-001 treatment. (**d**,**f**) Changes in LDL-C and HDL-C 3 h after CER-001 injection. (**g**,**h**) Triglyceride levels during CER-001 treatment. A paired *t*-test was performed to compare TC, LDL-C, HDL-C, and triglyceride values between 0 and 3 h after CER-001 injection, **** *p* < 0.0001 and ns *p* > 0.05.

Different studies have reported that in addition to decreased HDL-C levels, the entire lipid profile is altered in severe COVID-19. Indeed, both total cholesterol, HDL-C, and LDL-C levels are decreased in bacterial sepsis as well as in severe COVID-19. Here, we show that total cholesterol levels were not significantly impacted by CER-001 injections (Figure 1a,b) and that LDL-C levels were low but in the normal range at admission (1.92 mmol/L) and fell to minimal values at D3 (0.33 mmol/L). LDL-C increased slightly after the 3rd to 6th injections of rHDLs and returned to basal concentrations during the patient’s worsening phase (from D4 to D7) (Figure 1c), but without a significant impact of rHDL injections (Figure 1d). At admission, HDL-C levels were normal (1.26 mmol/L). Interestingly, HDL-C decreased 3 h after each rHDL injection, suggesting that these particles were functional in terms of reverse cholesterol transport (Figure 1e,f). Noteworthy, the injected rHDL particles (CER-001) do not contain cholesterol and are composed only of phospholipids and ApoA1. Given that HDL-C concentration is determined by quantification of the cholesterol contained in the non-apoB lipoproteins, the reduction in HDL-C levels following rHDL injections may be due to an improved cholesterol transport to the liver and subsequent elimination. Triglyceride levels increased during the hospitalization period and fell after the patient’s condition deteriorated (Figure 1g). CER-001 had no significant effect on triglyceride levels 3 h after injection (Figure 1h).

### 2.3. Lipoprotein Particle Size Analysis by Lipoprint

**Figure 2:**
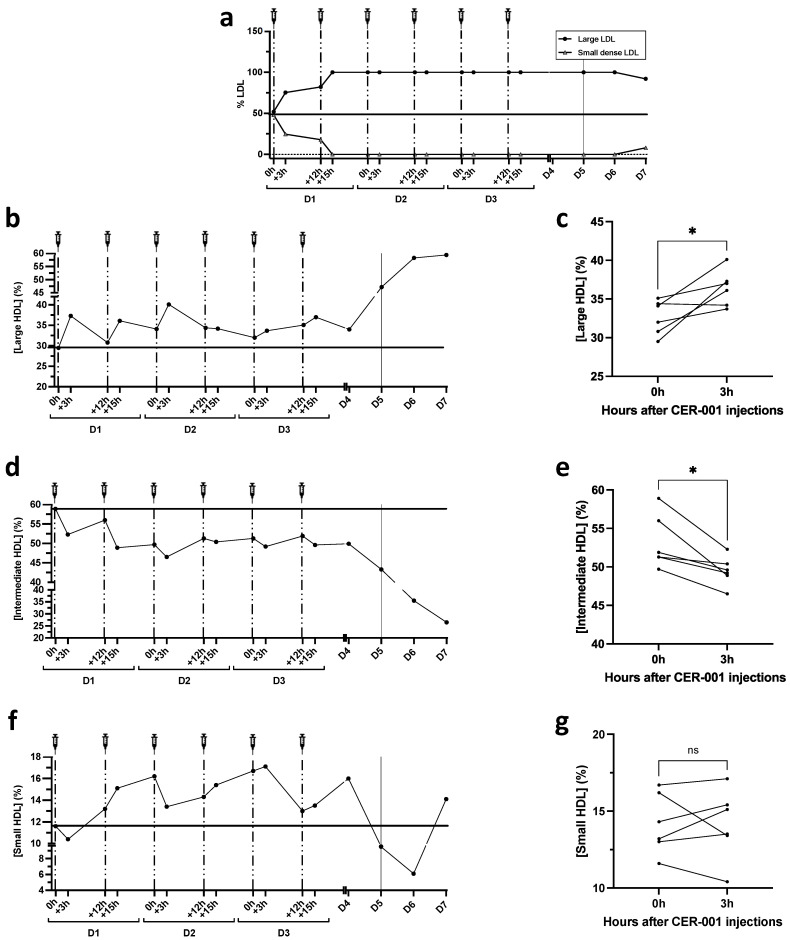
Changes in lipoprotein particle size during CER-001 treatment as determined by Lipoprint analysis. LDL and HDL subclasses were separated from 25 μL of plasma by non-denaturing non-reducing electrophoresis according to particle size. (**a**) Lipoprint analysis of small and large LDL particles. After the first 2 injections of CER-001, small LDL particles were no longer detectable during the rest of the treatment period (100% of large LDL particles). (**b**–**g**) Lipoprint analysis of HDL fractions. Kinetics of HDL particle size changes during CER-001 treatment, showing (**b**) large, (**d**) intermediate, and (**f**) small HDL fractions. Comparison before and 3 h after CER-001 injections (**c**,**e**,**g**). A paired *t*-test was performed to compare the percentages of large HDLs, intermediate HDLs, and small HDLs before (0 h) and 3 h after CER-001 injection, * *p* < 0.0332 and ns *p* > 0.05.

The size of lipoprotein particles is often correlated with their properties and/or functionality. LDL and HDL particle size was assessed by nondenaturing, nonreducing electrophoresis (Lipoprint technique, Quantimetrix, Redondo Beach, CA, USA). The Lipoprint analysis was consistent with the U-shaped curve of LDL-C levels, with a significant decrease, especially of small LDL particles, that was almost undetectable after the first rHDL injections, to rise weakly at the critical phase, outside the period of rHDL injection (D5–7) (Figure 2a). More interestingly, we show an increase in the proportion of large HDL particles 3 h after CER-001 injection, in parallel with a decrease in intermediate-sized particles (Figure 2b–e). This result is expressed as % HDL-cholesterol and reflects the distribution of HDL particles of different sizes containing Sudan black-stainable lipids (native rHDL are poorly stained by this dye). The increase in large HDL particles reflects the ability of injected rHDL to accept and transport cholesterol/cholesterol esters. It is consistent with the decrease in total HDL-C levels observed 3 h after rHDL injection, suggesting their maturation and increased catabolism (hepatic clearance).

Although non-significantly different after repeated injections of rHDL, the percentage of small HDL particles was slightly increased during the first 3 days and an inverse trend paralleled the worsening of the patient’s condition on days 5, 6, and 7 (Figure 2f,g). These results are consistent with previous studies that report that small HDL particles are more antioxidant and anti-inflammatory, whereas large HDL particles are less protective.

### 2.4. Apolipoprotein Concentration in Plasma

**Figure 3:**
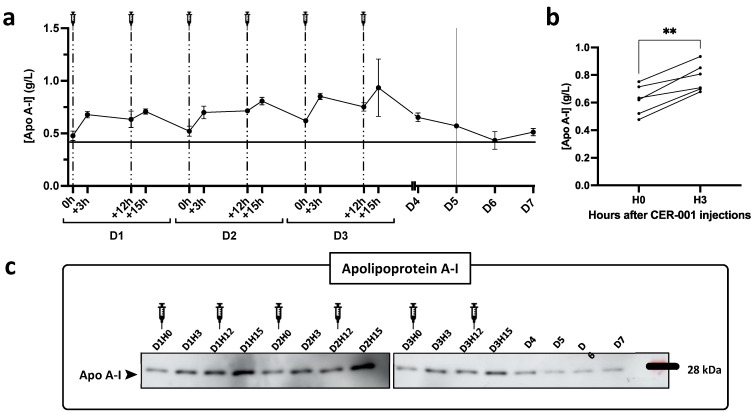
Plasma apolipoprotein A-I (Apo A-I) levels following patient treatment with CER-001. Apolipoprotein A-I concentrations were measured in plasma diluted 1:40,000 (Mabtech Inc ELISA kit, 3710-1HP2, Nacka Strand, Sweden) as detailed in Supplementary File S1. The patient received two injections of CER-001 per day over three days with a 12 h interval between each injection represented by the syringes and dashed lines. On day 5, the patient enters a critical phase represented on the graph (**a**) by a continuous line at D5. Plasma was collected before (0 h, +12 h) and 3 h after (+3 h, +15 h). Apolipoprotein A-I levels were assessed in patient plasma by ELISA (**a**,**b**) and Western blot (**c**). (**a**) Plasma was diluted 1:40,000. Apolipoprotein A-I concentration is provided in g/L. Each point represents the mean ± SD of 3 independent experiments. (**b**) Comparison of ApoA1 levels before and 3 h after CER-001 injections. ** *p* < 0.01. (**c**) Western blot analysis of patient plasma at all time points. Plasma was diluted 1:200 and 8 μL were analyzed with an antibody specific for apolipoprotein A-I, to confirm the ELISA results.

Given that rHDLs consist of recombinant apoA1 and phospholipids, we tested for potential changes in the patient’s plasma apolipoproteins (including apoA1) that might reflect lipoprotein remodeling during and outside the period of injections. ApoA1 levels were determined by three different techniques: ELISA, Western blot (Figure 3), and mass spectrometry, which also allowed quantification of various other apolipoproteins (Apo(a), ApoA-II, Apo A-IV, Apo B100, Apo(a), Apo C-I, Apo C-II, Apo C-III, Apo D, Apo E, Apo H, Apo J, Apo M, Apo L1). As expected, plasma apoA1 increased after the different injections to reach a maximum concentration 3 h after the 4th and 5th injections (0.80 and 0.85 g/L). The same trend was observed by ELISA and Western blot, as well as by mass spectrometry. The plasma concentration of apolipoproteins other than ApoA1 did not vary significantly after the rHDL injections.

### 2.5. Shotgun Proteomics on Isolated HDL Particles Following CER-001 Injections

**Figure 4:**
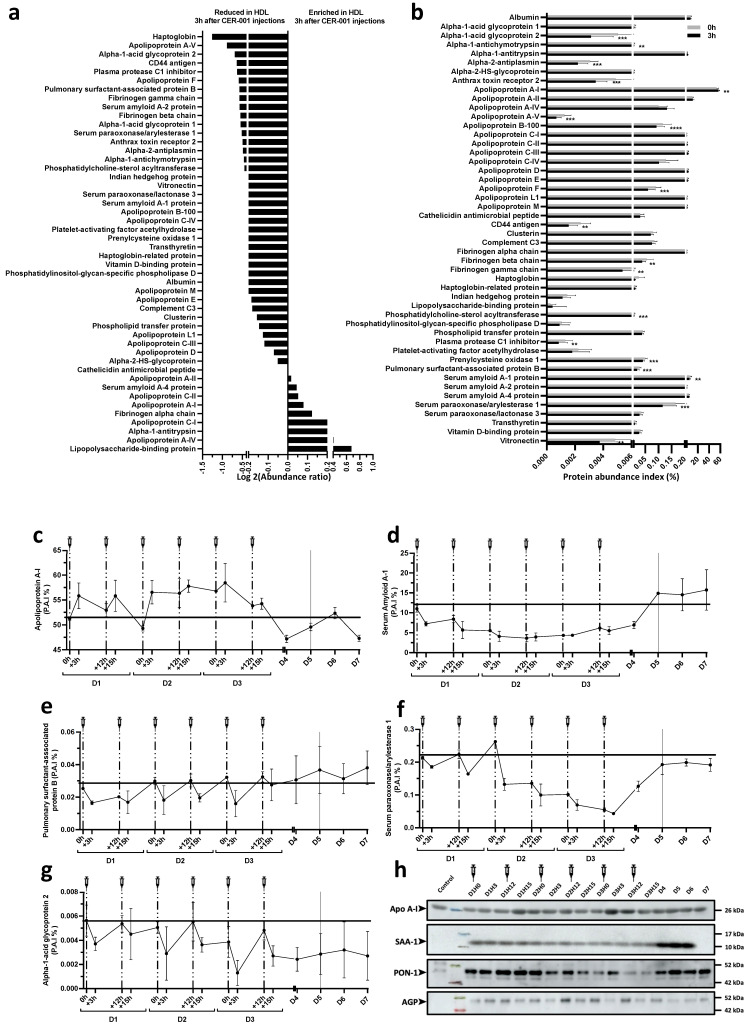
Change in HDL protein cargo during CER-001 treatment. The patient’s HDLs were isolated by ultracentrifugation. In total, 5 μg of patient HDL were alkylated, reduced, digested with TPCK-trypsin, and acidified. The digested peptides were then recovered, desalted with PierceTMpeptide desalting spin columns, and dried. Peptides were resuspended in 20 μL of ACN with 0.05% TFA and then analyzed by online nano-LC using an Ultimate 3000 NCS-3500 RS system coupled with a nanospray-ionization mass spectrometer (NSI)-Q-Orbitrap (Q Exactive Plus, Thermo Fisher Scientific, Bremen, Germany). Protein identification and quantification were performed using Proteome discoverer software. The entire method is described in Supplementary File S1. Shotgun analysis by nano-LC-MS/MS was performed on HDL preparation at all time points. (**a**) Changes in relative HDL protein abundance at 0 and 3 h after CER-001 injections were expressed as Log 2 (abundance ratio) (3 h/0 h). (**b**) Variations in HDL protein abundance 3 h after CER-001 injection. Protein abundance is defined as the protein abundance index (P.A.I.) expressed as a percentage. The bars in the graph represent the mean of percent intensity ± SD. A multiple paired *t*-test was used with the two-step post hoc method of Benjamini, Krieger, and Yekutieli with FDR set at 1% to adjust the *p*-value. ** *p* < 0.0021, *** *p* < 0.0002, **** *p* < 0.0001 compared with 0 h after CER-001 injection. Percentage change in apolipoprotein A-I (Apo A-I) (**c**), serum-amyloid A-1 (SAA-1) (**d**), pulmonary surfactant-associated protein B (**e**), paraoxonase/arylesterase-1 (PON-1) (**f**), and alpha-1-acid glycoprotein-2 (AGP-2) (**g**) during and after CER-001 treatment. (**h**) Western blot analysis HDL at all time points. A total of 5 μg of HDL per lane was transferred and analyzed with specific antibodies for apolipoprotein A-I, SAA-1, PON-1, and acidic alpha-1-glycoprotein (AGP).

Whereas total plasma apolipoproteins were not significantly altered, we hypothesized that CER-001 injections may result in changes in the HDL proteome. Shotgun proteomics was performed by LC-MS/MS after trypsin digestion of 5 micrograms of HDLs isolated by ultracentrifugation as described previously [37] and detailed in Supplementary File S1. The results presented in the Figure 4 were generated from the same amount of total protein initially digested before mass spectrometry analysis. Additional normalization was performed by assuming that the total amount of peptides present in each sample was the same. ApoA1 was more abundant in HDLs isolated 3 h after CER-001 injections whereas most of the other differential proteins identified were less abundant (Figure 4a,b). These proteins include Alpha-1 acid glycoprotein 2, Apha-1 antichymotrypsin, Alpha-2 antiplasmin, Anthrax toxin receptor 2, Apo A-V, Apo B-100, Apo F, CD44 antigen, Fibrinogen alpha and gamma chain, Phosphatidylcholine-sterol acyltransferase, Plasma protease C1 inhibitor, Prelycysteine oxidase 1, Pulmonary surfactant-associated protein B, Serum paraoxonase/arylesterase 1, and Vitronectin. These results suggest that rHDL supplementation results in the formation of ApoA1-enriched HDLs, whose turnover may be increased, promoting the clearance of proteins associated with normal or pathological HDL particles such as pulmonary surfactant-associated protein B or SAA-1 (Figure 4c–g). As shown in Figure 4h, Western blot analysis globally confirmed the results obtained by mass spectrometry. For instance, SAA-1 was significantly less abundant in HDL particles isolated during the CER-001 treatment period. This hepatic acute phase protein was abundant in HDLs isolated from the patient at admission, and began to decrease after the 2nd injection, reaching its minimal levels at D2–3, after the 3rd to 5th injections. HDL-SAA increased significantly at D4, 5, 6, and 7, paralleling the deterioration of the patient.

### 2.6. Assessment of Plasma Inflammatory Markers Following CER-001 Injections

**Figure 5:**
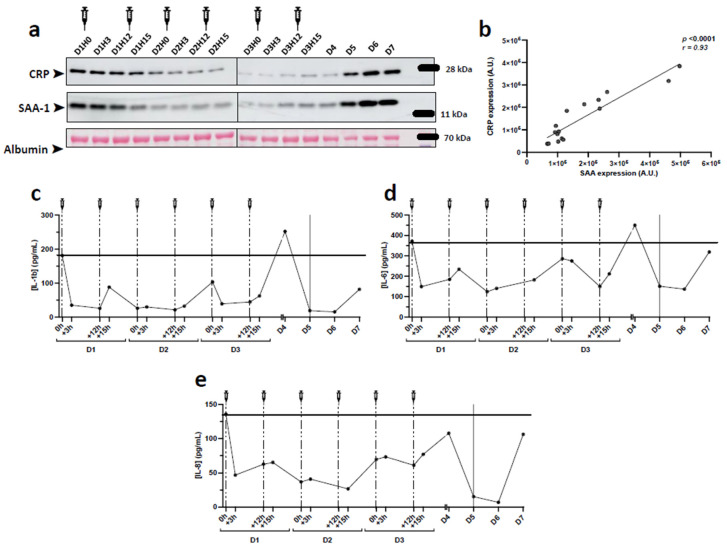
Markers of inflammation in plasma after CER-001 injections. The levels of IL-1b, IL-6, and IL-8 were quantified from 1:2 diluted plasma using Luminex xMAP technology. Cytokine concentrations were determined using the Human Custom ProcartaPlex 12-plex kit. The method is detailed in Supplementary File S1. (**a**) Western blot analysis of acute phase proteins in the patient’s plasma. Plasma was diluted 1:40,000, separated to constant volume (8 μL), and analyzed with antibodies specific for C-reactive protein (CRP) and serum amyloid A-1 (SAA-1) after blotting. (**b**) Positive correlation between CRP and SAA-1 after quantification of Western blots (r = 0.93, *p* < 0.0001, Pearson correlation). IL-1b (**c**), IL-6 (**d**), and IL-8 (**e**) concentrations were determined by Bioplex from 1:2 diluted plasma.

SAA-1 is an acute phase protein reported to replace ApoA1 and to render HDLs dysfunctional and pro-inflammatory. Given that under inflammatory conditions, SAA-1 is primarily associated with HDLs, we tested whether HDL-SAA-1 could reflect its plasma levels. As shown in Figure 5a by Western blot on plasma samples, SAA-1 showed the same trend as in isolated HDL particles. On admission, SAA-1 levels were elevated and decreased during the rHDL injection period, only to increase again during the deterioration phase. Similarly, C-reactive protein (CRP), which is not associated with HDLs, showed the same trend and its abundance correlated well with that of SAA-1 (Figure 5b). Assessment of inflammatory cytokines and other markers by multiplexed ELISA showed a significant decrease for most of them at D2 before a peak was observed at D4 when the patient’s condition deteriorated, especially for IL-6, IL-1beta, and IL-8 (Figure 5c).

## 3. Discussion

Beyond the initial phase of viral infection by SARS-CoV-2, the host response is characterized by an inflammatory storm mediated by the release of cytokines and acute phase proteins that participate in potential clinical complications. Numerous molecular targets have been proposed and a plethora of potential therapeutic agents have emerged to combat this post-infection phase that is critical for the clinical outcome of patients [48]. Oxidative stress is also a hallmark of SARS-CoV-2 pathogenesis and antioxidant therapies have, thus, been proposed to alleviate COVID-19 complications [49]. The pleiotropic properties of high-density lipoproteins (HDLs), in addition to their lipid transport function, include anti-inflammatory, antioxidant, anti-thrombotic, and overall endothelial protective effects [50] that may be beneficial in mitigating the inflammatory storm characterizing COVID-19. In addition, as previously reported for bacterial sepsis, HDL-cholesterol levels are markedly decreased in COVID-19 patients and may be related to disease severity [32,33,51]. Supplementation with reconstituted or recombinant HDLs (rHDLs, respectively made with apoA1 isolated from human plasma or by genetic engineering) have shown significant anti-inflammatory effects both in vitro and in vivo, in preclinical or clinical studies. In humans, several studies have reported the safety of intravenous injections of rHDLs in different clinical settings ranging from diabetes [52,53] and dyslipidemia [54] to atherosclerosis [40]. In the present study, we sought to investigate for the first time the inflammatory and lipoprotein profiles of a patient with severe COVID-19 who received six intravenous injections of rHDLs (CER-001, Abionyx Pharma) in an intensive care unit, benefiting from a temporary authorization for use.

On admission, the lipid profile was normal, with low LDL-C levels, but within the normal range. LDL-C levels decreased significantly during the period of rHDL infusion. In a previous study using the same source of rHDL (CER-001) in familial hypoalphalipoproteinemia (FHA), the authors showed a slight decrease in LDL-C levels 1 h after injection followed by an increase in non-esterified cholesterol and a decrease in esterified cholesterol maintained for 8 h [55]. Here, the decrease in LDL-C cannot be attributed solely to rHDL injections but rather to COVID-19 severity, as reported in several studies [35,56,57]. Furthermore, we show that HDL-C was significantly decreased 3 h after CER-001 injections, whereas ApoA1 concentration was increased. This is not in line with previous studies showing an increase in both HDL-C and ApoA1 after injection of rHDLs (CSL111) in diabetic and FHA subjects (CER-001) [55,58]. Here, it can be hypothesized that there is an increase in the clearance of HDL-C particles, as shown in bacterial sepsis, resulting in a decrease in HDL-C. In addition, the reduced cholesterol efflux capacity could participate in this decrease in HDL-C, as described in endotoxemia [59]. However, in our study, Lipoprint analysis demonstrated an increase in large size HDL particles, suggesting that injected CER-001 may have matured into large HDLs. In mice, injection of rHDLs (CSL-111) induced an increase in HDL particle size with cholesterol enrichment, suggesting remodeling due to increased cholesterol efflux capacity [60]. Enrichment of large HDL particles was reported in bacterial sepsis [21] and a decrease in small, pre-beta HDL particles was observed by NMR in endotoxemia [59].

In the present study, we sought to assess the composition of HDLs after isolation by ultracentrifugation, the standard reference method used to study these lipoproteins. Under inflammatory conditions, including COVID-19, the composition of HDLs is profoundly altered. We and others have previously shown that SAA (serum-amyloid A)-containing HDLs are dysfunctional and exhibit attenuated endothelial protective effects, particularly under inflammatory conditions, such as COVID-19 [37]. Here, we show that at admission, this acute-phase protein reported to replace ApoA1 in HDLs was highly abundant relative to control HDLs isolated from healthy subjects in which SAA-1 was almost undetectable. HDL-SAA decreased after rHDL injections to minimal levels at D2–3 after the 4th and 5th injections, and then increased significantly again at D4, 5, 6, and 7, reflecting the deterioration of the patient. It is likely that the last injection of rHDLs (D3H12) was not sufficient to bind and eliminate SAA-1, which is abundantly secreted by the liver into the plasma. Except for ApoA1, all other HDL-associated proteins, including those related to inflammation, were decreased after injection of CER-001, suggesting improved clearance of pro-inflammatory HDL particles and potentially a better bioavailability of functional ApoA1 nanoparticles. Indeed, even if after injection, HDL particles are mainly enriched in ApoA1, which alone exerts most of the pleiotropic protective properties attributed to HDLs [50]; this may produce beneficial effects in the context of COVID-19.

In agreement with our previous publication [37], a recent proteomic study reported that HDL cargo was profoundly altered with COVID-19 severity [61], with increased abundance of several proteins such as SAA-1 and -2, SFTPB, ApoF, and inter-alpha-trypsin inhibitor heavy chain H4 while ApoM was more associated with survival in these patients. In the present study, SAA-1, SFTPB, and ApoF were significantly decreased in HDLs 3 h after CER-001 injections. SAA-1 renders HDL pro-inflammatory by replacing ApoA1 and by reducing their cholesterol efflux capacity [62]. SFTPB is a specific marker of the lung proteome, capable of binding HDLs and altering their antioxidant function [63]. Increased clearance of these proteins may be beneficial in COVID-19.

We have previously reported that injection of rHDLs (CSL-111) in three different models of bacterial sepsis in mice reduced inflammation and limited mortality [28]. We also demonstrated that in severe COVID-19, HDLs from patients had reduced anti-inflammatory effects [37]. Here, we tested the overall inflammatory burden reflected in plasma by cytokine production as well as secretion of C-reactive protein and SAA-1 (mainly associated with HDLs). Plasma CRP followed the same trend as SAA-1 and confirmed a correlation between the patient’s severity status and acute phase hepatic protein levels.

The multiplexed ELISA analysis performed on inflammatory markers showed a decrease for most of them at D2, but without significant effects 3 h following CER-001 injections. This may be due to the kinetics of production and cellular origin of these cytokines, which does not allow an immediate impact in plasma a few hours after HDL infusions. An increase in IL-6, IL-1beta, and IL-8 was observed at D4 when the patient deteriorated. It appears premature to conclude that the cessation of HDL infusion was the cause of this increase in pro-inflammatory cytokines, also reflected by the increase in CRP and SAA (in plasma and both plasma and HDL for the latter).

About 30% of patients admitted in ICU for severe COVID-19 die despite therapeutics. These therapies combine supportive care (sedation, neuromuscular blockade, mechanical ventilation, catecholamines, renal replacement therapy) and until recently, only steroids have shown efficacy in severe COVID-19 pneumonia. In this case report, several explanations can be given such as an immediately refractory respiratory state on admission to hospital preventing curative action of therapies (corticoids and CER-001) and a respiratory condition weakened by COPD and overweight which is an unfavorable factor in COVID-19. Even if it is difficult to draw any conclusion with this case report, it could have been opportune to continue the CER-001 after day 3 when the patient clinically deteriorated from day 4. Only a randomized, controlled, double-blind study evaluating CER-001 infusion could be helpful in this context.

The limitations of our study are as follows:

- we report only one case of a patient who received an injection of rHDL.
- the decrease in inflammation after rHDL administration cannot be attributed solely to this innovative treatment, due to the lack of statistics.
- Moreover, as recommended, the patient received corticosteroids for 7 days since her arrival in ICU, which may obviously interfere with our results. While corticosteroids were continued, the increase in proinflammatory parameters after stopping rHDL infusion nevertheless raises questions.

In conclusion, our case report shows for the first time that intravenous HDL supplementation is feasible in acute inflammatory conditions such as COVID-19, with a tendency to limit inflammation. HDLs have been shown to reduce mortality in preclinical models of bacterial sepsis via their ability to bind and remove circulating lipopolysaccharides. However, in viral sepsis, less is known about the potential mechanisms by which HDL infusion may be beneficial, particularly by reducing inflammation and via their endothelial protective effects. This case report encourages us to carry out a randomized placebo-controlled trial to evaluate the contribution of rHDL in severe ICU COVID-19 patients.

## Figures and Tables

**Table 1:** Laboratory test of the patient during ICU hospitalization.

Laboratory Tests	During CER-001 Treatment												After CER-001 Treatment			
	Day 1				Day 2				Day 3				Day 4	Day 5	Day 6	Day 7
	H0	H3	H12	H15	H0	H3	H12	H15	H0	H3	H12	H15				
Leukocytes (×10^9^/L)	5.41		5.67		9.17		10.47		8.62		10.48		9.88	20.74	19.39	36.64
Neutrophils (×10^9^/L)	4.07		4.55		7.08		8.97		6.41		8.76		7.68	18.67	17.01	31.07
Hemoglobin (g/dL)	13.8		13.7		12.7		13.1		11.7		12.6		11.8	11.7	11.4	12.1
Platelets (×10^9^/L)	217		250		265		298		212		273		255	173	255	278
Protein (g/L)	75	77	76	71	76	68	73	64	64	66	71	64	65	68	63	64
Creatinine (µmol/L)	41	43	43	47	43	43	45	44	43	45	40	36	41	49	46	73
AST (U/L)	44	49	46	45	46	54	63	52	54	60	76	63	65	58	34	65
ALT (U/L)	59	62	67	63	67	72	83	74	77	82	112	97	101	108	87	127
Lactates (mmol/L)	0.9	0.9	1		0.9	1	1.2		1	1.3	1.3		1.1	1.1	1.3	3.8
CRP (mg/L)	147	124	110	98	72	70	62	48	33	36	48	54	65	202	315	265
Total cholesterol (mmol/L)	3.78	4.62	4.32	4.45	3.85	4.71	5.24	5.04	4.92	5.70	5.65	5.48	5.59	4.02	4.47	4.98
HDL-C (mmol/L)	1.26	0.86	1.03	0.75	0.94	0.64	0.97	0.74	1.02	0.69	1.17	0.80	1.12	1.44	0.78	0.74
Triglycerides (mmol/L)	1.34	2.19	2.61	3.11	3.81	4.11	4.72	4.47	4.82	5.28	5.39	4.69	5.47	2.01	2.60	3.44
LDL-C (mmol/L)	1.92	1.89	1.21	1.05	0.59	0.74	0.44	0.48	0.33	0.57	0.31	0.43	0.37	1.04	1.82	2.33

ALT: alanine aminotransferase; AST: aspartate aminotransferase; CRP: C-reactive protein; HDL-C: high-density lipoprotein cholesterol; LDL-C: low-density lipoprotein cholesterol.

## Data Availability

Data sharing not applicable.

## Supplementary Materials

The following supporting information can be downloaded at: https://www.mdpi.com/article/10.3390/biomedicines10040754/s1, Supplementary File S1: methods used in the manuscript. References [37,64,65] are cited in the supplementary materials
