# Supplementary material for: First Recombinant High-Density Lipoprotein Particles Administration in a Severe ICU COVID-19 Patient, a Multi-Omics Exploratory Investigation
Source: Biomedicines. 2022 Mar 23;10(4):754. doi: 10.3390/biomedicines10040754 (PMC9029957; doi:10.3390/biomedicines10040754)
Supplement: Supplementary file 1 [file biomedicines-10-00754-s001.zip › biomedicines-1611709-supplementary.pdf]

Plasma analyses

Various analyses were performed directly on plasma (including lipid profile, cytokine quantification and proteomics for detection of C-Reactive protein, Apo A1 and other apolipoproteins) or on HDL particles after isolation by ultracentrifugation. In particular, HDL proteome modifications were assessed by shotgun mass spectrometry. The size of different lipoprotein particles was evaluated by non-denaturing, non-reducing electrophoresis and Sudan black staining (Lipoprint technique).

Quantification of lipoprotein subpopulations by electrophoresis (Quantimetrix Lipoprint LDL)

Two LDL subpopulations were separated by size using the Quantimetrix Lipoprint LDL kit (Quantimetrix Corporation, Redondo Beach, CA, USA). The Lipoprint LDL is a test validated by the Food and Drug Administration (March 2002, FDA) for the measurement of lipoprotein cholesterol subpopulations for research and clinical diagnostic purposes. This kit is based on the separation of lipoprotein cholesterol subpopulations in a precast 3% native polyacrylamide gel electrophoresis tube [1]. The experiment and analyses were performed according to the manufacturer's instructions. Briefly, 25 µL of plasma were mixed with 200 µL of loading buffer at the top of the gel tube. The loading gel containing the sample was photopolymerized for 35 min at room temperature. The gel tubes were then electrophoresed at 3 mA/gel. Electrophoresis was stopped when the HDL fraction was 1 cm from the end of the tube (±1 h). The tubes were allowed to rest in the dark for 30 minutes. The gel tubes were then scanned and analyzed using Lipoware LDL software. The percentage of cholesterol was determined in lipoproteins (VLDL to HDL) including 7 subpopulations of LDL, separated by size. The subpopulations of LDL-1 to 2 correspond to large LDLs, and the subpopulations of LDL-3 to 7 correspond to small dense LDLs. Then, the percentage of LDL was calculated using this equation:

$$\%LDL = \frac{\%\ subpopulation\ LDL\ (sdLDL\ or\ large\ LDL) \times 100}{\sum \%\ subpopulations\ LDL}$$

Similarly, HDL subpopulation were quantified using Quantimetrix system. Lipoprint HDL tube assay and analysis were performed according to the manufacturer's protocol. Briefly, 25 µL of EDTA plasma were mixed with 300 µL of loading gel and this mixture was placed on top of the gel tube before photopolymerization for 35 min at room temperature. Then, electrophoresis of the gel tubes was performed at 3 mA/gel and until the albumin fraction reached 1.5 cm from the bottom of the tube (± 50 min). After allowing the tubes to rest for 30 min in the dark, they were scanned and analyzed using Lipoware software. After analysis, ten HDL subpopulations were measured and corresponded to three

HDL subpopulations (HDL-1 to HDL-3: large, HDL-4 to HDL-7: intermediate, and HDL-8 to HDL-10: small HDL particles. These three major families were quantified and expressed as a percentage of total HDL cholesterol.

Measurement of plasma apolipoprotein A-1 concentrations by ELISA

Plasma apolipoprotein A-I concentrations were determined by ELISA (Mabtech Inc, 3710-1HP2) directly from total plasma (1:40 000) according to the manufacturer's recommendations. ELISA was performed in three independent experiments for all patient collection times.

Quantification of cytokines by multiplex immunoassay

Plasma samples were analyzed using Human Custom ProcartaPlex 12-plex (Affymetrix, eBioscience, Vienna, Austria) according to the manufacturer's instruction. Procartaplex uses Luminex xMAP technology for quantification of 12 cytokines: CD31, CD40, CD62E, Caspase-3, E-Cadherin, ICAM-1, IL-1b, IL-6, IL-8, PAI-1, VCAM-1, and tPA. Plates were read by the Bio-Plex 200 (Bio-Rad) and cytokine concentrations were calculated according to standard curves using the Bio-Plex Manager software (version 6.2, Bio-Rad laboratories).

Apolipoprotein quantification by mass spectrometry

Plasma preparation for mass spectrometry analysis

Seventeen apolipoproteins and LCAT were quantified by a liquid chromatography-mass spectrometer (LC-MS/MS) as described previously [2] with some modifications. Briefly, unlabeled proteotypic peptides and isotope-labeled analogs from Thermo Scientific Custom peptide synthesis service (Scotland, United Kingdom) were resuspended at 1 mM in 50% ACN with 0.1% formic acid (FA). A pool of these unlabeled peptides was serially diluted in distilled water to obtain a range of seven standards. Isotope-labeled peptides (i.e., "heavy" amino acid containing [$13C_6$, $15N_2$] K, [$13C_6$, $15N_4$] R, and [$13C_6$, 15N] I at the C-terminal position) were used as internal standards (ISs). A stock solution of mixed ISs (32 µM) was diluted in digestion buffer (50 mM ammonium bicarbonate). Subsequently, each sample was spiked to a final concentration of 1.6 µM.

Plasma samples were prepared with the ProteinWorksTM eXpress kit (Waters, Milford, MA, USA) following the kit protocol. Protein denaturation was performed using 30 µL of plasma (1:3 dilution), mixed with 20 µL of RapidGest SF (High) surfactant solution (7 mg/mL), and incubated at 80°C for 10 min. Samples were reduced with 8.75 mM dithiothreitol for 10 min at 60°C, alkylated with 15.7 mM iodoacetamide for 30 min in the dark, and finally digested with 140 µg of TPCK (L-1-tosylamido-2-phenylethyl chloromethyl ketone trypsin)-treated trypsin overnight (16h) at 37°C. The resulting tryptic

peptides were acidified with 2% TFA and incubated for 15 min at 45°C to stop enzymatic digestion. The tubes were centrifuged for 45 min at 14,000 g. The supernatant was collected and cleaned on the Oasis HLB 1 cc, 30 mg vac cartridge (Waters, part no. WAT094225). Catridges were preconditioned and equilibrated before loading the samples (180 µL). Peptides were then washed with 5% methanol (1 mL), eluted with 80% methanol (500 µL), and dried under nitrogen flow. Then, peptides were resuspended in 150 µL of 5% ACN and 0.1% FA for mass spectrometry analysis.

Shotgun LC-MS/MS analysis

Samples were separated by ultra-high-performance liquid chromatography coupled with a Q-Orbitrap mass spectrometer (Q Exactive Plus, ThermoFisher, Waltham, MA, USA). Briefly, 30 µL of sample were injected using an UHPLC system equipped with a Thermo Fisher Ultimate 3000 series WPS-3000 RS autosampler and separated on an Aeris® PEPTIDE XB-C18 column (1.7 µm, 100 mm × 2.1 mm, Phenomenex, Torrance, CA USA). Peptides were eluted on three linear elution gradients, starting with 1% to 8% solution B (0.1% formic acid in 100% ACN) until 1.50 min, then 8% to 29% solution B for 8.50 min, and 29% to 95% solution B for 1 min. The column was then washed for 2.30 min with 95% solution B and equilibrated with 1% solution B for 2 min. A blank containing solution A (0.1% formic acid in 5% acetonitrile) was added every 10 samples. The flow rate was set at 350 µL/min and the column temperature was held at 60°C. For mass spectrometer parameters, the heated electrospray ionization (HESI) was set at 3.5 kV, the capillary temperature was set at 300°C, and the S-lens RF was set at 50%. Samples were analyzed by a targeted mass spectrometry approach by using the parallel reaction monitoring (PRM) mode. The PRM acquisition method combined several time-scheduled targeted MS/MS events. MS/MS spectra were acquired at a resolving power of 17,500 (at *m/z* 200) with automatic gain control (AGC) set to 2e5 and a maximum injection time (max IT) of 120 ms. The targeted peptides were isolated using a 3.0 *m/z* window and fragmented by higher energy collisional dissociation (HCD) with an adapted normalized collision energy comprised between 16 and 35 eV (Supplemental material Table 2). Elution and quantification of apolipoproteins were performed by two separates runs to avoid co-elution and to increase sensitivity, accuracy and precision. PRM data were processed in skyline software (version 21.1.0.146, MacCoss Lab, Seattle, WA, USA). Quantification of apolipoproteins and LCAT was calculated by plotting the peak intensity ratios of the unlabeled peptide (sum of light transitions) to isotope-labeled peptide (sum of heavy transitions) versus analyte concentrations with linear regression (1/X weighting, linear through zero). The calibration curve of each peptide had a correlation coefficient (R^2) of 0.966-1.

Lipoprotein isolation

All subsequent steps were performed under a class II microbiological safety cabinet. Lipoproteins were separated from EDTA plasma by two-step ultracentrifugation as previously described [3]. Briefly, the density of the plasma was adjusted to 1.063 using potassium bromide (KBr) solution with a density of 1.35 (500 µL of plasma was mixed with 99 µL of KBr d=1.35). Plasma with a density of 1.063 was overlaid with 1.3 mL of d= 1.063 KBr solution in 2mL Ultraclear tubes. Ultracentrifugation was performed on a Beckman Coulter Optima L-80XP at 252,000 g for 20 h at 10°C in a 50.4 Ti rotor. The lipoprotein fraction at the top of the tube containing LDL was collected after the first ultracentrifugation. KBr from this first lipoprotein fraction was then removed by 4 wash steps with EN saline buffer (500 µL, 0.9% NaCl, 1 mM EDTA, and 0.025% NaN3, pH 7.8) in a centrifugal filter device (Amicon Ultra, 3 KDa cut off, UFC500396). For isolation of HDLs, the lower fraction obtained after the first ultracentrifugation was recovered (150 µL) and the density was adjusted to 1.21 with KBr solution (525 µL, d=1.35), and then, deposited under 1 mL of KBr solution d=1.21. After the second ultracentrifugation (252,000 g for 20 h at 10°C), HDL was recovered, washed with the same procedure as for the LDL fraction, and concentrated to 100 µL in EN saline. The protein concentration of HDL was determined by the conventional bicinchoninic acid technique (BCA, Sigma) according to the manufacturer's instructions. HDL purity was estimated after separation in 12% SDS-PAGE and coomassie blue staining, showing the absence of contaminating proteins.

HDL proteome analysis by mass spectrometry

The preparation of HDLs and the nano LC-MS/MS analysis were performed in triplicate for the different samples.

Preparation of HDL for mass spectrometry analysis

HDL protein concentration was determined by BCA (Sigma) using bovine serum albumin as a standard range. Sample preparation was performed as previously described [3] with some modifications. Briefly, 5 µg of HDL was added to 25 µL in digestion buffer (6M urea 50 mM ammonium bicarbonate) and denatured at 80°C for 10 min. The samples were then reduced and alkylated. After alkylation, four volumes of 50 mM ammonium bicarbonate were added to the samples to dilute the urea. HDLs were then digested overnight at 37°C with 3 µg of TPCK-trypsin (Sigma). Samples were acidified with 2% trifluoroacetic acid (TFA) and incubated for 15 min at 4°C to inhibit trypsin digestion. Then, the supernatant containing the digested peptides was recovered after 15min of centrifugation at 10,000 rpm at 10°C. The peptides were then desalted on Pierce^{TM}peptide desalting spin columns (Thermo Fisher Scientifics, No. 2162704) and eluted with 50% ACN 0.1% TFA and dried by Speed Vacuum.

Shotgun nanoLC-MS/MS analysis

The dried peptides were resuspended in 20 µL of 2% ACN with 0.05% TFA, and analyzed by online nano-LC using an Ultimate 3000 NCS-3500 RS system coupled with a nanospray-ionization mass spectrometer (NSI)-Q-Orbitrap (Q Exactive Plus, Thermo Fisher Scientific, Bremen, Germany). Briefly, 5 µL of samples were injected into a C18 LC-EASY-spray column (2 µm, 100 Å, 75 µm × 50 cm, Thermo Fisher Scientific). Peptides were eluted on two linear elution gradients starting from 4 to 25% solvent B (0.1% formic acid in 80% ACN) until 103 min, and then from 25 to 40% solution B for 20 min. A washing step was then carried out with 90% solution B for 5 min followed by an equilibration step with 4% solution B for 20 min. The column was then washed for 5 min with 90% solution B and equilibrated with 4% solution B for 20 min. Solution A was 0.1% formic acid in water. To avoid carry-over between samples, two blanks containing acidified water were performed between each sample. The flow rate was set at 0.300 µL/min and the column temperature was held at 40°C. For mass spectrometry parameters, the NSI was set at 1.8 kV, the capillary temperature was set at 275°C, and the S-lens RF level was set at 30%. Mass spectra were recorded in data-dependent acquisition from *m/z* 350 to 2,000 in positive ion mode. Full scan MS spectra were acquired at a resolving power of 70,000 (at m/z 400) with automatic gain control (AGC) set to 5e5 and a maximum injection time (max IT) of 100 ms. MS/MS spectra of the ten most intense precursor ions were acquired at a resolving power of 35,000 (at *m/z* 400) with an AGC set to 1e5 and a maximum IT of 100 ms. The normalized collision energy was set to 28 eV for peptide fragmentation. Precursor ions were dynamically excluded within 60 seconds to avoid repeated peptide selection.

Protein identification and quantification of shotgun nanoLC-MS/MS data

Protein identification and quantification were performed automatically from the Xcalibur raw files using Proteome discoverer software (version 2.2.2.2.0). The MS and MS/MS spectra were matched against the Uniprot human reference proteome database with only canonical sequences (26 559 sequences; 01 July 2021) with the SEQUEST HT search engine. The search in SEQUEST HT was performed with the following parameters: oxidized methionine and protein N-terminal acetylation were set as variable modifications and carbamidomethylation of cysteines as fixed modification. The specific enzyme was trypsin and two missed cleavages were allowed. A mass tolerance of 10 ppm was used for the precursor ions with 0.02 Da. The false-discovery rate (FDR) was fixed to 1% at the level of proteins and peptides using a target-reversed decoy database search strategy. A minimum of one unique peptide sequence with a Sequest score (Xcorr) ≥ 2 was used. Label-free quantification was performed on identified proteins by using the peak intensities of validated peptides for a given protein. Unique peptides were used for quantification.

Data processing

All analyses were performed on Proteome Discoverer software. The missing value of each protein was replaced by the 25 percentiles of the three independent experiments for each sample. Protein abundance index (PAI) was defined as the percentage of the intensities of one protein over the sum of the intensities of all proteins whose identification was manually verified by interpretation of the corresponding MS/MS spectra. Protein groups correspond to the average protein abundance index for all samples in each group (0h vs. 3h). The 0h group corresponds to the time before CER-001 injection and the 3h group corresponds to the sample 3h after CER-001 injection. The abundance ratio was defined as the ratio of the DPI in the 3h group to the DPI in the 0h group. Log 2 (abundance ratio) was calculated to show the variation 3h after CER-001 injections.

Western Blot

Western Blot of plasma

SDS-PAGE (12% polyacrylamide) allowed separation of proteins from plasma (8 µL of a 1:200 dilution), followed by transfer to a nitrocellulose membrane as described previously [3]. After transfer, the presence of proteins was verified by Ponceau red staining. The following antibodies were used: apolipoprotein A-I (Origene, AP14057PU-N used at 1:10,000), serum amyloid A-1 (Abcam, ab190802, used at 1:10,000), C-reactive protein (Sigma, 128K45734, used at 1:5,000).

Western blot of HDLs

Five µg of HDL were electrophoresed as described for plasma and then transferred to a nitrocellulose membrane using a semidry transfer at 90 mA for 1.5 hours (Hoefer Scientific Instruments, San Francisco, USA). The following steps of blocking, primary and secondary antibodies, and washing are the same as for the plasma western blot. The antibodies used were: apolipoprotein A-I (Origene, AP14057PU-N used at 1:10,000), serum amyloid A-1 (Abcam, ab190802, used at 1:10,000), alpha-1-acid glycoprotein (Abcam, ab134042, used at 1:1,000).

Statistical analysis

Statistical analyses were performed with Prism 9 software (GraphPad Software Inc., San Diego, CA, USA). The p values were considered significant when $p < 0.05$. The paired t-test was performed to compare the 0h vs 3h group after CER-001 injections. Pearson correlation was used for parametric evaluation between SAA and CRP expression. A multiple paired t-test with the two-step post-hoc method of Benjamini, Krieger, and Yekutieli with FDR set at 1% to adjust the p-value was applied to compare PAI between the 0h vs 3h group after CER-001 injections.

Bibliography

1. Hoefner, D.M.; Hodel, S.D.; O'Brien, J.F.; Branum, E.L.; Sun, D.; Meissner, I.; McConnell, J.P. Development of a Rapid, Quantitative Method for LDL Subfractionation with Use of the Quantimetrix Lipoprint LDL System. *Clin Chem* **2001**, *47*, 266–274.
2. Blanchard, V.; Garçon, D.; Jaunet, C.; Chemello, K.; Billon-Crossouard, S.; Aguesse, A.; Garfa, A.; Famchon, G.; Torres, A.; Le May, C.; et al. A High-Throughput Mass Spectrometry-Based Assay for Large-Scale Profiling of Circulating Human Apolipoproteins. *J Lipid Res* **2020**, *61*, 1128–1139, doi:10.1194/jlr.D120000835.
3. Begue, F.; Tanaka, S.; Mouktadi, Z.; Rondeau, P.; Veeren, B.; Diotel, N.; Tran-Dinh, A.; Robert, T.; Vélia, E.; Mavingui, P.; et al. Altered High-Density Lipoprotein Composition and Functions during Severe COVID-19. *Sci Rep* **2021**, *11*, 2291, doi:10.1038/s41598-021-81638-1.
